# Ketogenic diet and ketamine infusion treatment to target chronic persistent eating disorder psychopathology in anorexia nervosa: a pilot study

**DOI:** 10.1007/s40519-022-01455-x

**Published:** 2022-08-23

**Authors:** Lori Calabrese, Barbara Scolnick, Beth Zupec-Kania, Caroline Beckwith, Kayla Costello, Guido K. W. Frank

**Affiliations:** 1 Innovative Psychiatry, South Windsor, CT USA; 2 Internal Medicine and Addiction Medicine, Waban, MA USA; 3 Ketogenic Diet Therapy, Elm Grove, WI USA; 4 Clinical Peer Counselor, New York, NY USA; 5 Center of Excellence in Eating and Weight Disorders, Icahn School of Medicine at Mount Sinai, New York, NY USA; 6 Department of Psychiatry, University of California San Diego, San Diego, CA USA

**Keywords:** Anorexia nervosa, Eating disorders, Ketamine, Ketamine treatment, Keto diet, Ketogenic diet

## Abstract

**Purpose:**

Anorexia nervosa (AN) is a severe psychiatric disorder, and shape and weight concerns are often chronic despite weight normalization. No specific treatments exist for those preoccupations that interfere with recovery and trigger relapse. A case study using a ketogenic diet followed by ketamine infusions led to sustained remission in one patient with chronic AN. Here we conducted an open-label trial to test whether this response could be replicated.

**Methods:**

Five adults weight recovered from AN but with persistent eating disorder thoughts and behaviors adopted a therapeutic ketogenic diet (TKD) aimed at maintaining weight. After sustaining nutritional ketosis, participants received six ketamine infusions and were followed over 6 months.

**Results:**

All participants completed the study protocol without significant adverse effects. Two participants maintained TKD for 8 weeks prior to ketamine infusions due to good behavioral response and remained on TKD. Three participants received TKD for 4 weeks prior to and during ketamine, then tapered off after the final infusion. The group showed significant improvements on the Clinical Impairment Assessment (*p* = 0.008), Eating Disorder Examination Questionnaire (EDEQ) Global score (*p* = 0.006), EDEQ-Eating Concerns (*p* = 0.005), EDEQ-Shape Concerns (*p* = 0.016), EDEQ-Weight Concerns (*p* = 0.032), Eating Disorders Recovery Questionnaire (EDRQ) Acceptance of Self and Body (0.027) and EDRQ-Social and Emotional Connection (*p* = 0.001). Weight remained stable, except for one participant who relapsed 4 months after treatment and off TKD.

**Conclusion:**

This novel treatment appears to be safe and effective for adults with chronic AN-related psychopathology. The results from this open trial support that there are specific neurobiological underpinnings of AN that can be normalized using TKD and ketamine.

**Level of evidence:**

Level IV, multiple time series with intervention

**Supplementary Information:**

The online version contains supplementary material available at 10.1007/s40519-022-01455-x.

## Introduction

Anorexia nervosa (AN) is a severe psychiatric disorder, typically presenting at puberty, marked by self-starvation, fear of weight gain, distortions in body image, and compulsions regarding food and exercise. AN often becomes a chronic and enduring illness that is driven by persistent distress over weight gain and body image. The mortality rate of AN is among the highest among the psychiatric disorders, but there is no FDA-approved medication for the treatment of AN, and the effectiveness of psychotherapeutic interventions especially for severe and enduring AN is limited [1].

The etiology of AN remains unclear. Recent research suggests biological mechanisms that contribute to AN’s pathophysiology [2]. For instance, the activity-based anorexia (ABA) animal model showed that a high-fat low-carbohydrate diet both prevented self-starvation and rescued rodents who had begun to self-starve and run excessively [3]. During a high fat low carbohydrate diet, the organism utilizes ketone bodies for energy generation, suggesting that this mechanism could be useful in treating AN. In another ABA paradigm, the single injection of the N-methyl-d-aspartate (NMDA) receptor antagonist ketamine was protective for survival [3], and infusion of ketamine in adult women with chronic AN led to improvement in obsessional symptoms [4]. Both ketogenic diet and ketamine are modulators of the dopamine circuitry**.** Dopamine may have a central role in the pathophysiology of AN [5], and ketogenic diet and ketamine could help normalize altered dopamine function in AN. For instance, ketogenic diet affects dopamine metabolism and has been shown to protect dopaminergic neurons against neurotoxicity [6]. Ketamine, on the other hand, leads to dopamine release [7], which may reset a circuitry that has adapted to food restriction by low dopamine release but elevated receptor sensitivity and high anxiety. Ketogenic diet has been shown effective in neurological conditions and shows promise in mood disorders, and could similarly help normalize brain circuit function and behaviors in AN [8].

Previously, we published a case report about a 29-year-old woman with chronic AN who adopted a therapeutic ketogenic diet (TKD) similar to that used to treat epilepsy and not designed for weight loss [9]. Within a few days on the treatment the AN-specific symptoms improved. Ketamine infusions were added as the TKD alone was not sufficient to attain complete remission. After nutritional ketosis for 2 months, followed by four ketamine infusions, she experienced a complete remission from AN [9]. From a body mass index (BMI) of 18.2 at treatment begin, she gained 15 pounds over several months and returned to her pre-morbid weight. She remains on the TKD and in complete remission at 28 months (personal communications).

To investigate whether we would find evidence that TKD and ketamine would be safe and effective in the treatment of AN-specific psychopathology such as eating, shape and weight concerns, we conducted a follow-up trial in a larger group. Due to concerns of the potential of weight loss during TKD, we recruited individuals with a diagnosis of AN who were weight recovered but had significant elevations in AN-typical behaviors. We hypothesized that similar to the first patient on TKD and ketamine, we would find substantial improvement in AN-related psychopathology.

## Methods

### Participants

Females over the age 18 years with history of AN for at least 10 years were recruited from the community through targeted advertisements. The study was approved by IntegReview Institutional Review Board, Texas, USA, and written informed consent was obtained from all participants. Participants were not compensated. The trial was registered with ClinicalTrials.gov NCT 04714541.

Potential participants were screened for eligibility by completing an online screening form, phone interview, and then video interview with the principal investigator. Eligible participants had a physical examination with their primary care physician, resting electrocardiogram, and laboratory screening including electrolytes, glucose, aspartate aminotransferase, alanine transaminase, calcium, magnesium, phosphorous, complete blood count, prealbumin, lipid panel, acylcarnitine panel, serum pregnancy test, urinalysis, and urine tox screen. Laboratory values had to be within normal range for study admission. Participants continued current psychiatric medications under the care of their outpatient provider. Of the 25 adults who were screened initially, 5 met criteria for participation.

A psychiatric interview (LC) assessed for psychiatric diagnoses and verification of AN with weight recovery. Diagnosis of AN was met using criteria from the Diagnostic and Statistical Manual of Mental Disorders (DSM-5) and was confirmed with the Eating Disorder Assessment of DSM-5 Feeding and Eating Disorders (EDA-5) Version 3.0 (Adult). Participants also completed the Eating Disorder Examination Questionnaire (EDE-Q), Clinical Impairment Assessment (CIA) for Eating-Disorders, Eating Disorders Recovery Questionnaire (EDRQ) and the Patient Health Questionnaire-9, (PHQ-9) (see supplement for detailed information).

### Design and procedure

The trial sequence consisted of a therapeutic ketogenic diet (TKD), designed by an experienced ketogenic dietitian which involved a 2-day immersion program, home maintenance of nutritional ketosis for 4–8 weeks, followed by six titrated ketamine infusions. TKD was aimed at inducing nutritional ketosis but not losing weight.

### Study settings

Immersive introduction to the clinical trial and the TKD occurred in person over a 2-day period for the whole group. Maintenance of TKD and nutritional ketosis occurred at home. Administration of ketamine infusions occurred in an outpatient private psychiatric clinic.

### Immersion and maintenance of TKD

Each participant was accompanied by an adult support person who was a family member or friend. Immersion into nutritional ketosis using a therapeutic TKD focused on the following: encouraging three meals and two snacks daily of ketogenically balanced macronutrients of which fat would provide the main source of calories, followed by protein then carbohydrate. The macronutrient ratio of the therapeutic TKD used in this study was 70% fat, 20% protein and 10% carbohydrate. During the 2-day immersion program, all study staff (including psychiatrist, internist, experienced ketogenic dietitian/nutritionist, and peer support counsellor), participants, and their support persons met informally, prepared all meals together, and ate together. The patient who was the index case from our previous case report was present throughout and offered peer support.

Breath acetone is an accepted non-invasive indication of ketosis, although there is not direct equivalence between breath acetone and blood ketones. Each participant was provided with Keyto (getkeyto.com), a commercial breath acetone meter, and measured breath acetone twice daily for 2 weeks, then daily for the remainder of the TKD phase of the study. The Keyto monitor reports levels on a 0–6 + scale, and the goal for each participant was to achieve and maintain levels of at least 4 throughout the active intervention phases of TKD and ketamine administration.

Participants were blinded to weight measurements, which were recorded daily for 1 week, every other day for 1 week, then weekly until study completion and obtained by a medical provider, the participant’s support partner, or ClearStep^®^, a scale without visible display that transmitted data directly to the research team.

Following the immersion program, participants returned home and were monitored for safety, TKD adherence and efficacy with daily phone calls, weight, and breath acetone monitoring, which tapered to biweekly monitoring by the end of four weeks. Weekly optional group meetings were offered by study staff for additional monitoring and support. After four weeks of demonstrated nutritional ketosis, participants could elect to continue the TKD for an additional 4 weeks before proceeding to ketamine administration.

### Ketamine administration

Each participant received a total of six infusions of racemic ketamine within a 17-day period, modified from the planned 3-week period due to participant transportation and vacation plans. The initial dose of 0.75 mg/kg body weight and titration goal of 1.2 mg/kg administered over 45 min, was modeled after the index patient to accomplish dissociation [9]. Pulse, blood pressure, respiration, and O_2_ saturation were monitored throughout the infusion and recovery periods.

Participants were given ondansetron orally dissolving tablets 8 mg before each infusion. Nausea and dizziness were managed respectively with intravenous metoclopramide 2.5 mg or oral meclizine 25 mg. Participants were prepared to expect dissociation by the psychiatrist.

### Follow-up

After the sixth ketamine infusion, follow-up assessments were conducted weekly for 4 weeks, monthly for 3 months, then every 3 months until 6 months with plans to continue follow-up again at months 9 and 12. For this report (Table 1) baseline is coded as T0, T1 indicates 4 weeks after starting TKD, T2 indicates 4 weeks TKD + 4 weeks Ketamine for participants 1, 2, 5 or 8 weeks TKD only for participants 3, 4; T3 indicates 1 month post ketamine, T4 indicates 3 months post ketamine, T5 indicates 6 months post ketamine. Each remote follow-up evaluation included objective body weight measurement, questionnaire responses, detailed qualitative interviews about participant reported outcomes including personal responses to the intervention, TKD adherence/liberalization, eating disorder symptoms, behaviors, presence of the “anorexic voice” and when possible, interviews with participants’ treatment teams and support persons. Here we report on outcome data up to month 6.

**Table 1:** Repeated measures ANOVA for primary behavioral outcome measures, as well as body mass index, BMI

	T0	T1	T2	T3	T4	T5	Part. η^2^	Power	*F*	*p*	*p* (Huyn-Feldt)
Clinical impairment assessment	35.40 ± 4.72	28.60 ± 11.41	18.40 ± 14.33	18.80 ± 10.45	15.40 ± 9.61	13.00 ± 12.02	0.517	0.896	4.276	0.008	0.008
EDEQ global	3.14 ± 1.36	2.44 ± 1.18	2.48 ± 1.22	1.58 ± 0.88	1.12 ± 0.49	0.95 ± 0.62	0.534	0.918	4.587	0.006	0.024
EDEQ restraint	2.65 ± 1.15	1.28 ± 0.90	1.88 ± 1.78	1.20 ± 1.14	0.49 ± 0.32	0.65 ± 0.49	0.401	0.689	2.673	0.052	0.098
EDEQ eating concern	2.12 ± 1.48	1.64 ± 1.49	1.32 ± 1.12	0.72 ± 0.30	0.28 ± 0.33	0.38 ± 0.30	0.539	0.924	4.682	0.005	0.006
EDEQ shape concern	4.12 ± 1.63	3.96 ± 1.41	3.78 ± 1.60	2.50 ± 1.64	2.14 ± 1.16	1.86 ± 1.09	0.479	0.839	3.672	0.016	0.025
EDEQ weight concern	3.64 ± 1.69	2.84 ± 1.53	2.88 ± 2.01	1.98 ± 1.62	1.66 ± 0.58	0.94 ± 0.40	0.435	0.760	3.083	0.032	0.077
EDRQ lack of symptomatic behavior	5.16 ± 0.45	5.38 ± 0.22	5.46 ± 0.50	5.42 ± 0.54	5.70 ± 0.25	5.48 ± 0.45	0.155	0.212	0.735	0.606	0.567
EDRQ acceptance of self and body	1.38 ± 1.05	2.06 ± 0.94	2.36 ± 1.66	3.12 ± 1.24	3.42 ± 1.15	3.62 ± 0.96	0.446	0.781	3.225	0.027	0.027
EDRQ social and emotional connection	3.32 ± 0.44	3.22 ± 0.84	3.48 ± 0.97	3.78 ± 0.56	4.76 ± 0.52	5.04 ± 0.26	0.636	0.989	6.977	0.001	0.001
Patient health questionnaire	14.00 ± 3.94	10.80 ± 6.61	9.60 ± 5.68	8.80 ± 5.59	7.40 ± 6.99	8.20 ± 6.14	0.338	0.556	2.046	0.115	0.120
Body mass index (kg/m^2^)	21.02 ± 3.14	20.74 ± 2.68	20.50 ± 2.41	20.38 ± 2.45	20.68 ± 2.51	20.10 ± 2.57	0.280	0.431	1.552	0.219	0.272

Mauchly’s test for sphericity could not be calculated due to the small sample size and the Huynh–Feldt *p* value correction was added in case of violations of data sphericity

*EDEQ* Eating Disorder Examination Questionnaire; *EDRQ* Eating Disorders Recovery Questionnaire

### Statistical analysis

Data were analyzed using SPSS 28 (IBM.com) using descriptive statistics as well as repeated measures ANOVA. False discovery rate (FDR) was used to control for multiple comparisons of behavioral data.

## Results

### Demographic and clinical characteristics

All participants were white females, ages 29–45 (M38.4 ± 7.4) years. All had completed college, three were married and two had children. All participants had been diagnosed with AN-restricting type, now weight restored but with ongoing severe AN-related preoccupations. Participant characteristics with age, current comorbidity, medications and final ketamine dose: P1: Age 32 years; major depressive disorder (MDD), posttraumatic stress disorder (PTSD); 1.3 mg/kg; P2: Age 45 years, MDD; vilazodone, gabapentin; 1.1 mg/kg; P3: 42 years, MDD, obsessive compulsive disorder (OCD), body dysmorphic disorder, PTSD; sertraline; 1.2 mg/kg; P4: 44 years; MDD, generalized anxiety disorder (GAD); 0.75 mg/kg; P5: 29 years; MDD, GAD, panic disorder; fluoxetine, brexpiprazole, lisdexamfetamine, dextroamphetamine; 1.3 mg/kg.

### Ability to adopt and maintain nutritional ketosis with TKD

All participants maintained nutritional ketosis for at least 4 weeks before receiving ketamine infusions. Three participants achieved nutritional ketosis by the end of the 2-day immersion program (breath acetone levels above 5); a fourth participants achieved ketosis within the first week; one participant required an additional 4 weeks to fully adopt TKD and achieve sustained nutritional ketosis. After maintaining nutritional ketosis on TKD for at least 4 weeks, two participants elected to continue TKD with breath acetone monitoring for an additional 4 weeks before ketamine administration; after the sequenced intervention ended and follow-up began, they both opted to maintain the TKD, with some liberalization, throughout follow-up.

### Adverse effects

Participants did not report significant unexpected adverse events during the immersion, adoption and home adherence to TKD, or during the weeks when participants maintained TKD and were receiving ketamine infusions. Specifically, no patient reported suicidal ideation.

### Outcome data

Participants largely provided outcome data but participant 4 did not provide EDEQ data on time point 2 (8 weeks) and participant 1 at time point 5 (6-month follow-up). Participant 5 did not complete EDRQ data on time point 2, participant 4 on time point 3, and participant 1 on time point 5; CIA data were not available for participant 4 for time point 1, participant 5 for time points 2 and 3, and participant 1 for time point 5. PHQ data were missing for participant 5 for time points 2 and 4, and participant 1 for time point 5. Last observation carried forward (LOCF) was used to handle missing data as is commonly applied in longitudinal studies. BMI data were available for all participants for all time points.

**Fig. 1:**
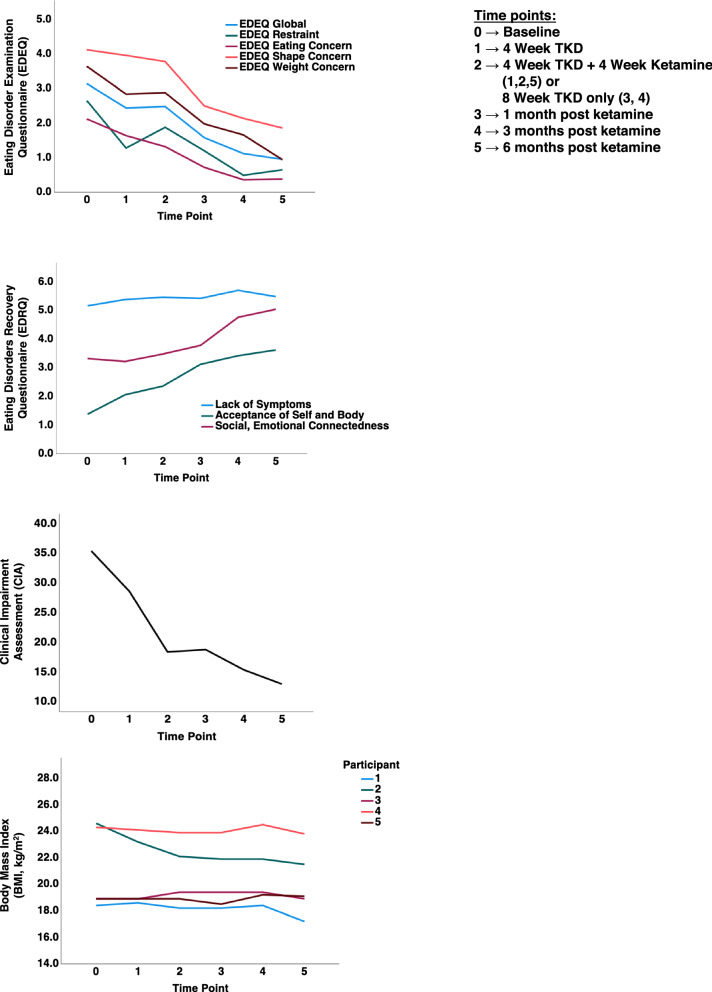
Graphical representation of composite values for behavioral measures and body mass index (BMI) over time

There were significant effects for the scores over time for the CIA, EDEQ Global, EDEQ Eating Concern, EDEQ Shape Concern, EDRQ Acceptance of Self and Body and EDRQ Social and Emotional Connection (Table 1; Fig. 1).

BMI did not significantly change over time. However, participant 1 trended lower 4 months after the last ketamine infusion and was admitted to a residential program.

Participant individual feedback is provided in the supplemental material.

## Discussion

This follow-up study on the previously published case report indicates that TKD followed by ketamine infusion treatment normalizes AN-related psychopathology. Weight remained largely stable in this cohort and the treatment did not result in any severe adverse effects that would have required stopping the trial.

All study participants reported improvement after TKD and more so after addition of the ketamine intervention. This raises important mechanistic questions. Genetic studies implicate central neurotransmitter function as well as metabolic mechanisms that contribute to AN [2]. Ketogenic diet provides an alternative energy source for cellular function and protects dopaminergic neurons against neurotoxicity [6]. Ketamine affects dopaminergic neurotransmission and normalizes circuits associated with emotional processing [7, 8]. The underlying mechanisms due to TKD and ketamine that contribute to reversal of AN-related behaviors in this sample cannot be determined by this study. However, they are intriguing and require further study. During TKD, the brain shifts from relying on glucose as the exclusive source in “usual” circumstances, to utilizing ketones to supply 60% of the energy requirement. Changing the brain’s available energy source changes brain physiology and functioning, implicating effects on mitochondria, lipids as signaling agents, adenosine, neuronal stabilization due to less fluctuations in glucose, and changes in the gut microbiome [10]. It is possible that an alternative energy source is beneficial to reduce AN-related symptoms. However, nutritional ketosis also is associated with reduced inflammatory markers, which could contribute to the findings.

While the study sample was small, there were significant changes observed in symptom improvement, with large effect sizes. For some individuals, TKD provided significant relief from AN behaviors to the extent that they desired to stay longer on TKD. However, in all cases, the addition of ketamine provided additional relief, suggesting that there was no ceiling effect. One participant relapsed 4 months after the intervention and required a higher level of care. That individual did not remain on TKD and it can be speculated whether she would have remained significantly improved had she stayed on the ketogenic diet. It is possible that for some long-term TKD is therapeutic. Two individuals remained on TKD through the 6-month follow-up and have remained largely symptom free.

### Strengths and limits

This well-designed study indicates that TKD plus ketamine is beneficial to reduce AN-related psychopathology. All participants were diagnosed with comorbid MDD, while some had anxiety, OCD- or trauma-related disorders. Whether TKD or ketamine had specific effects on depression that subsequently improved AN-related psychopathology, or whether the effects were independent require further study. The sample size was small, participants were permitted to extend the 4-week adherence to TKD to 8 weeks before ketamine infusions, and to taper/discontinue the TKD following the final infusion. Ketamine dose was titrated and variable, and integration of the dissociative experience was variable. Other limitations include the lack of a control group, and the use of breath acetone as an approximation of ketosis rather than blood betahydroxybutyrate.

## Conclusions

These results of this study suggest that TKD that is aimed to establish and maintain nutritional ketosis but not weight loss, followed by ketamine infusion treatment, is safe, effective and can provide symptomatic relief for individuals with AN who have weight normalized but continue to have severe preoccupations with fears of weight gain, body shape concerns and difficulties with self-acceptance. These results are promising and support larger studies to identify novel interventions to promote lasting recovery.

## What is already known on this subject?

Animal research implicated ketogenic diet and ketamine as potentially beneficial to normalize brain dysfunction and behaviors in models for anorexia nervosa. A case report in a person with long-standing anorexia nervosa showed sustained recovery after ketogenic diet and ketamine treatment.

## What this study adds?

This study adds to the evidence that ketogenic diet and ketamine may be generalizable to individuals with anorexia nervosa in reducing symptoms. The results of the study support the need for larger studies in anorexia nervosa to test the effectiveness of ketogenic diet and ketamine.

## Supplementary Information

Below is the link to the electronic supplementary material.

Supplementary file1 (DOCX 28 KB)

## Data Availability

The datasets generated or analyzed during the current study are available from the corresponding author on reasonable request.
