# Supplementary material for: Ketogenic diet and ketamine infusion treatment to target chronic persistent eating disorder psychopathology in anorexia nervosa: a pilot study
Source: Eat Weight Disord. 2022 Aug 23;27(8):3751–7. doi: 10.1007/s40519-022-01455-x (PMC9803738; doi:10.1007/s40519-022-01455-x)
Supplement: Supplementary file 1 — Supplementary file1 (DOCX 28 KB) [file 40519_2022_1455_MOESM1_ESM.docx]

**Supplemental Material**

Assessments

Diagnosis of AN was met using criteria from The *Diagnostic and Statistical Manual of Mental Disorders* (DSM-5) and was confirmed with Eating Disorder Assessment of DSM-5 Feeding and Eating Disorders (EDA-5) Version 3.0 (Adult). Participants also completed the self-assessments *Eating Disorder Examination Questionnaire (EDE-Q),* a 28-item self-administered scale, which measures severity of eating disorder symptoms across the domains of dietary restraint, eating concern, weight concern, and shape concern, and also derives a global scale [1], *Clinical Impairment Assessment (CIA) for Eating-Disorders,* a 16-item self-administered scale, which measures severity of psychosocial impairment in domains of life affected by eating disorders [2], *Eating Disorder Recovery Endorsement Questionnaire (EDRQ,* a 28-item self-administered rating scale to assess lack of symptomatic behavior (LSB), acceptance of self and body (ASB), social and emotional connection (SEC), and physical health (PH) [3], and the *Patient Health Questionnaire-9,* (PHQ-9) to assess presence and severity of depressive symptoms [4]*.*

Participants Individual Information

Participant 1. remained on the TKD for four weeks before beginning ketamine infusions and tapered off the TKD after her sixth infusion. She had a minor response to diet alone, but a robust response to TKD+Ketamine, which started to wane between three- and six-month follow-up. At four-month follow-up she noted her food restriction was more pronounced. She had entered a residential treatment program during 6-months follow up.

Participant 2. remained on TKD for four weeks, followed by ketamine infusions and tapered off TKD after her sixth infusion. She did not report subjective noticeable changes in AN symptomatology during TKD alone. During and immediately post ketamine, she had a robust response, but at two-month follow-up, she experienced subjectively described “emotional overload” from the insights she experienced. She reported she needed to personally re-integrate the experience over several weeks, gather strength, and attain more freedom from the AN thoughts. At six-month follow-up, she reported ongoing marked improvement.

Participant 3. noted a marked decrease of symptoms on TKD alone. She opted to extend TKD to eight weeks prior to receiving ketamine. After receiving ketamine infusions, her AN symptoms continued to decline. At six-months she continued to maintain TKD.

Participant 4. reported subjective fast decline of AN symptoms on TKD and remained on TKD for 8 weeks. Added ketamine infusion added to symptom improvement, which she described as follows: "During the ketamine, I had a visual of a pile of circles, but the circles weren’t perfect and had rough edges. They were shaped in a pile in a pyramid, and they started crumbling and my brain thought ‘that’s my eating disorder and it is crumbling.’ It didn’t seem ‘super remarkable’ at the time but now it does." She maintained TKD at six-month follow up and considers herself in a strong recovery from AN. Her treatment provider who has followed her closely for over a decade, confirmed that perspective.

Participant 5. had initially low nutritional ketosis indicators. She was able however to achieve adequate ketosis followed by ketamine infusion, after which she tapered off and stopped TKD. She reported mild improvement of AN symptoms while on diet alone, which improved after ketamine infusions. She continues to have intrusive AN related thoughts.

**Supplement References**

1. Sysko R., Walsh B.T., Fairburn C.G.: Eating Disorder Examination-Questionnaire as a measure of change in patients with bulimia nervosa. Int. J. Eat. Disord., 37, 100-106, 2005.

2. Bohn K., Doll H.A., Cooper Z., O'Connor M., Palmer R.L., Fairburn C.G.: The measurement of impairment due to eating disorder psychopathology. Behav. Res. Ther., 46, 1105-1110, 2008.

3. Bachner-Melman R., Lev-Ari L., Zohar A.H., Linketsky M.: The Eating Disorders Recovery Questionnaire: psychometric properties and validity. Eat Weight Disord, 26, 2633-2643, 2021.

4. Levis B., Benedetti A., Thombs B.D., Collaboration D.E.S.D.: Accuracy of Patient Health Questionnaire-9 (PHQ-9) for screening to detect major depression: individual participant data meta-analysis. BMJ, 365, l1476, 2019.
